# How Coparenting Is Linked to Depression among Chinese Young Girls and Boys: Evidence from a Network Analysis

**DOI:** 10.3390/bs14040297

**Published:** 2024-04-04

**Authors:** Demao Zhao, Xin Gao, Wei Chen, Quan Zhou

**Affiliations:** 1 Collaborative Innovation Center of Assessment for Basic Education Quality, Beijing Normal University, Beijing 100875, China; 202031630013@mail.bnu.edu.cn (D.Z.); 201831630013@mail.bnu.edu.cn (X.G.); 2 School of Education, Tianjin University, Tianjin 300350, China; cw1109@tju.edu.cn; 3 Institute of Developmental Psychology, Faculty of Psychology, Beijing Normal University, Beijing 100875, China

**Keywords:** coparenting, depression, network analysis, young adolescents

## Abstract

This study aimed to explore the relationship between parental coparenting and depression among Chinese young adolescents and potential gender differences via network analysis. Thus, 793 fourth-grade students (girls: 281 (35.40%), Mage = 9.99 years, SD = 0.59 years) were recruited from three primary schools in Northern China. The young adolescents rated their depression and perceived paternal and maternal coparenting. Network analysis was used to detect the central nodes and bridge mechanisms among coparenting and depressive components. The results indicated that paternal and maternal consistency as well as maternal conflict were the most central components in the coparenting–depression network. Paternal consistency, maternal conflict and paternal disparagement in coparenting, as well as somatic complaints and positive affect in adolescents’ depression, exhibited high bridge strengths, suggesting those constructs served as vital bridges to connect the two subnetworks. Moreover, paternal consistency showed a higher bridge strength in the boys’ network than the girls’ one, whereas the edge linking adolescents’ positive affect to paternal disparagement and integrity was stronger in the girls’ network. This study contributes to the understanding of associations between parental coparenting and young adolescents’ depression and offered insights into targeted interventions for early adolescent depression by enhancing parental coparenting.

## 1. Introduction

Depression is one of the most prevalent mental health issues in modern society, profoundly impacting individuals’ psychological well-being and social functioning [1,2,3]. As unveiled by the World Health Organization (WHO), depression was ranked as the third-leading cause of global disease burden in 2004 and is anticipated to become the leading cause of mortality by 2030, significantly intensifying the global disease burden [4]. The early stage of adolescence is regarded as a critical juncture for the onset of depression, as adolescents entering this phase undergo a transformation featuring heightened sensitivity, bolstered self-esteem and a range of emotionally rich yet unstable states [5,6,7,8]. This renders them susceptible to external influences [8]. The findings of longitudinal studies indicate a rapid rise in the occurrence of depression during early adolescence compared to childhood [7,9]. Additionally, the initiation of depression for a majority of adults can be traced back to early adolescence [10,11]. Hence, it is significant to explore the factors and mechanisms underlying adolescent depression, especially in early adolescence, thereby providing effective interventions and therapeutic methods for adolescent depression [12]. 

Drawing from the bioecological model, parents play a crucial role within the family microsystem, dramatically affecting the developmental trajectory of children [13,14]. After birth, children gradually form a profound and long-lasting emotional bond with their parents through continuous interaction, leading to the establishment of parent–child attachment [15]. According to the classical attachment theory, the perceived attachment with parents significantly shapes children’s cognitive and emotional development [15,16]. Empirical studies indicated that children perceiving insecure parental attachment were more prone to exhibiting higher levels of depressive symptoms during adolescence [17,18]. In contrast, children with secure parental attachment were more likely to activate positive cognitive resources, attenuate negative emotions triggered by other events, thereby reducing the occurrence probability of adolescent depression [19]. Yet, parent–child attachment was originally constructed primarily through observations of interactions between mothers and children [20]. Moreover, in the traditional construct of the family, mothers typically shoulder the primary caregiving responsibilities, overseeing the majority of nurturing and upbringing tasks crucial for maintaining familial cohesion [21]. Conversely, fathers are commonly designated as the family’s financial backbone, with their contributions to parenting frequently relegated to the shadows [22]. This asymmetry in parental roles has led to a pronounced bias in research, with a predominant focus on exploring the impact of maternal parenting on adolescent depression [23,24]. However, this disproportionate emphasis on maternal influence may yield an incomplete understanding of familial dynamics [16,20,25,26]. Nonetheless, in the context of societal transformations, with mothers increasingly entering the workforce and fathers returning home to take on more childcare responsibilities, there has been a gradual equalization of parental roles between fathers and mothers within the family [27]. Consequently, the paternal influence on child rearing has gained increasing recognition [16,20,28]. Additionally, empirical research has also demonstrated that fathers and mothers played distinct roles and exhibited unique characteristics in their parenting approaches [29,30]. In recent years, the triadic interaction of fathers, mothers and children, known as the coparenting subsystem, in the family system has drawn widespread attention [31]. Coparenting refers to the collaborative activities among all adults in a family sharing parenting responsibilities during child upbringing [31,32], underscoring the interconnected interactions of individuals in their parental roles [33]. As suggested by family system theory, coparenting plays executive roles within the family dynamic as it serves as a common mechanism for regulating family boundaries and relationships [33]. Coparenting, as conceptualized by McHale [34], is a multidimensional construct that incorporates integrity, consistency, conflict and disparagement [31,35,36,37]. To be specific, integrity means the practices aiming to promote a sense of togetherness among family members [38,39]. Consistency is characterized as the alignment and endorsement of uniform disciplinary strategies between coparents [31]. Conflict is defined as the engagement of parents in arguments, disputes and physical confrontations in the presence of their children [40]. Disparagement refers to conveying belittling of the coparent and undermining the authority or credibility of the coparent when being alone with their children [36,37]. Within the coparenting framework, integrity and consistency are viewed as positive dimensions of coparenting, emphasizing fathers and mothers exhibiting supportive parenting behaviors that promote family cohesion and agreement to discipline their children. Conversely, conflict and disparagement are considered negative dimensions of coparenting, highlighting parents’ tendencies to display aggressive behavior or intentionally diminishing each other’s authority in coparenting children [31,32].

In family life, the quality of coparenting serves as a critical cornerstone influencing the socialization process of young adolescents [27,41,42]. Existing research has demonstrated that parental coordination, open communication and conflict resolution positively contribute to adolescents internalizing behavioral guidelines both at home and in school [43]. This, in turn, enhances the adaptive emotional regulation capacities of adolescents [41,44], bringing about a decline in adolescent depression [45,46]. In contrast, frequent and intense conflicts between parents often cause psychological distress in adolescents. A body of research has consistently revealed that adolescents exposed to negative coparenting practices are at a higher risk of developing both mental and physical health issues including depression [47,48,49,50,51]. Additionally, a project focusing on online intervention for coparenting conflict (the web-based Our Relationship program) conducted an intervention study with 213 couples who had at least one child aged 3 to 17 living with them. It was found that an improvement in relationship satisfaction among couples significantly reduced coparenting conflict, which, in turn, was strongly correlated with a reduction in children’s internalizing symptoms (such as depression). This underscores the significance of enhancing coparenting quality for lifting the psychological well-being of adolescents [52].

From the perspective of family systems theory, mothers and fathers play varying roles and have unique characteristics in the realm of coparenting, with mothering and fathering demonstrating interdependence in the family context [33,53]. For one thing, mothers and fathers may exhibit distinct coparenting attributes [33]. In detail, mothers typically take a more active role in educational and caregiving perspectives [54], whereas fathers are inclined to be more involved in recreational and leisure activities [55]. These distinctions may result in the development of distinct interactive relationships with children [33,56]. For another, a mutual interaction exists between maternal and paternal parenting, with the mood and parenting behaviors of one parent being transferred to the other [57]. In this way, the effects of coparenting behavior are not restricted to the self but also encompass the partner [27,30]. Hence, it is essential to assess both maternal and paternal coparenting behaviors to better capture the distinctions and interdependence of paternal and maternal coparenting on children’s depression [27]. Nonetheless, previous research on coparenting often utilized the combined coparenting behaviors of fathers and mothers as indicators, treating parents as a single entity [58] and failing to explore the distinctions in coparenting behaviors between fathers and mothers [59,60]. Alternatively, some studies only focus on paternal or maternal coparenting in separate models, without considering their interdependent effect on children’s mental health [36,61]. Notably, research on how both positive and negative coparenting practices from fathers and mothers affect adolescents’ depression is still relatively scarce. It has been challenging to apply traditional analytical methods to understand the underlying mechanisms between parental coparenting and adolescents’ depression. This is because traditional latent variable analysis methods might ignore the dynamic nature of the construction of parental coparenting (coparenting behaviors influence each other) [27,62] and also treat depression as a singular entity rather than a construction that comprises various interactive symptoms [63,64].

A new and promising method, which is called the “network analysis approach”, is increasingly being employed to probe into the relationships between psychological constructs and mental disorders in the psychopathology field [63,65,66]. The network analysis methodology predominantly relies on the Gaussian graphical model for constructing variable networks, where observed variables are represented as nodes, and the connections between nodes serve as edges [67]. Through the interconnection of nodes and edges, it visually elucidates the intricate relationships among variables. Furthermore, it assesses variable importance based on node centrality features, such as closeness, betweenness and node strength. The thickness and polarity (positive or negative) of edges are utilized to describe the partial correlations between variables [65,67,68]. Specifically, higher centrality in a node indicates the greater importance of the variable it represents in the relational network, and, once activated, nodes with high centrality can rapidly disseminate their influence throughout the entire network [69]. In addition, the strength of connections between nodes, often visualized with thick edges, indicates a closer relationship among variables [69,70]. The prominent advantages of this approach are that it conceptualizes psychological constructs as dynamic and primarily focuses on components that are measured by observed variables, rather than the whole latent constructs [66]. Further, within the framework of the network perspective on mental disorders, depression is understood not simply as a collection of isolated symptoms but as a complicated network of interconnected and interacting symptoms [71,72,73]. This paradigm shift is significant for the study and intervention of depression, as it highlights the causal relationships between symptoms and how these interactions contribute to the onset and maintenance of depressive disorders [71]. Particularly when it comes to exploring adolescent depression, the net-work analysis method has emerged as a powerful tool. It not only reveals the associations between specific symptoms but also helps us understand how various psychological and social factors exert an impact on the development of depression through these symptoms [71,72,73].

Moreover, research focusing on adolescent depression predominantly adopts the network analysis method to investigate the relationship between adolescent depression and peer relationships, childhood trauma, well-being, substance use and internet addiction. These studies shed light on the multifaceted influences on adolescent depression and how these factors play a role through the symptom network [74,75,76,77]. For instance, Wasil et al. [76] explored the relationship between well-being and the depression symptom network in a sample of Indian adolescents, clarifying that depressive symptoms are generally negatively partially correlated with well-being items, “feeling down about oneself”, in the depression symptom community and feeling cheerful in the happiness community bridged the symptom and happiness networks.

Despite the acknowledged significance of coparenting quality on young adolescents’ mental health [33,53], there is limited research utilizing network analysis to investigate the relationship between paternal and maternal coparenting behavior and young adolescents’ depression. By conceptualizing parental coparenting and young adolescents’ depression symptoms in the form of networks, one can identify which nodes are the most central in the network and which nodes serve as the bridges that link parental coparenting and youth depression [78,79]. This can provide valuable insights for strategic decision making, regarding which depression symptoms are the key symptoms to target for interventions and which coparenting behavior from mothers and fathers should be the primary target of the interventions to alleviate (or prevent) young adolescents’ depression.

Finally, adolescents’ gender deserves attention when exploring the relationships between coparenting behavior and youth depression [30,80]. Previous research has uncovered the significant role of gender in the adolescent attachment system, which can give rise to variations in how coparenting is perceived by boys and girls [30,81]. The spillover hypothesis from family systems theory suggests that the attachment dynamics formed in the parent–adolescent subsystem (father–adolescent and mother–adolescent attachment) may transfer into the coparenting subsystem and influence the quality of coparenting and, finally, the development of youth depression [82]. In light of this, the gender of adolescents may play a role in the interaction between parental coparenting and youth depression in nuanced and intricate ways that can be overlooked in the traditional ‘static’ approach [62]. In this context, the network approach can serve as a more appropriate methodology for gaining a profound understanding of gender disparities. Specifically, by employing the network comparison test (NCT), it becomes possible to discern whether the structures (i.e., connectivity patterns) and global strength (i.e., the sum of the absolute values of all edge weights in a network) of the models exhibit significant differences between boys and girls [68,71,83].

To sum up, the present study employed network analysis to examine the relationship between parental coparenting and depression symptoms in young Chinese adolescents and further compare gender differences in boys’ and girls’ networks. Specifically, it attempts to achieve two objectives. One is to examine the relationship between parental coparenting and adolescent depression within the total sample of adolescents, identifying the most central nodes in the total network and the bridge nodes linking parental coparenting and youth depression. The other is to compare the networks of boys and girls to clarify whether there are significant gender differences.

## 2. Materials and Methods

### 2.1. Participants and Procedure

A total of 793 fourth-grade students (girls: 281, M_age_ = 9.99 years, SD = 0.59 years) from three primary schools, including one urban, one suburban and one rural school, were recruited for the current study through convenience cluster sampling in Hebei Province, China. Written informed consent was obtained from both students and their parents before the formal tests. Participants were informed that participation was voluntary and that they were free to withdraw from the study at any time during data collection. Prior to completing the questionnaire, trained research assistants briefly introduced the purpose of data collection and provided guidance on survey completion. At the same time, participants were assured of the anonymity and confidentiality of their responses. The questionnaires took approximately 30 min to complete. The study was approved by the ethics committee of Beijing Normal University (Approval Number: 2022-46, Approval Date: 13 January 2022).

### 2.2. Measures

#### 2.2.1. Coparenting Scale

The Adolescence Revision of Coparenting Scale was used to evaluate maternal and paternal coparenting behaviors, as perceived by young adolescents [84]. This 29-item scale was based on Coparenting Scale [34] and was translated and revised by Chinese scholars Liu and colleagues [84]. This scale has demonstrated satisfactory reliability and validity [84] and has been widely adopted in studies investigating parental coparenting behaviors among young Chinese adolescents [27,84,85,86]. This scale encompasses four dimensions: integrity (e.g., “Father/Mother shows physical affection to mother/father”, “Father/Mother openly affirms or praises mother/father in my presence”, “Father/Mother facilitates enjoyable interactions between mother/father and me”; 7 items), consistency (e.g., “Father/Mother disciplines me in agreement with mother/father”, “When rewarding me, father/mother employs methods sanctioned by mother/father”, “When father/mother disciplines me, mother/father provides her/his support”; 10 items), conflict (e.g., “Father/Mother argues with mother/father”, “Father/Mother utters hurtful words about mother/father”, “Father/Mother criticizes mother/father in my presence”; 6 items) and disparagement (e.g., “Father/Mother criticizes mother/father when alone with me”, “Father/Mother enumerates mother/father’s shortcomings in my presence”, “When interacting with me, father/mother is critical of mother/father’s parenting approach towards me”; 6 items). Participants gave their responses for fathers and mothers using a 7-point Likert scale, ranging from “1 = never” to “7 = always”. Items were averaged, and higher scores reflected higher levels of paternal or maternal integrity, consistency, conflict and disparagement. Positive coparenting behavior was categorized by the dimensions of integrity and consistent behavior, whereas negative coparenting behavior was categorized by the dimensions of conflict and disparaging behavior. The Cronbach’s α regarding the four dimensions for paternal and maternal coparenting was 0.92, 0.93, 0.92, 0.95 and 0.92, 0.95, 0.92 and 0.97, respectively. In addition, the McDonald’s ω coefficient of the four dimensions was 0.92, 0.93, 0.92 and 0.96 and 0.92, 0.95, 0.92 and 0.96, respectively.

#### 2.2.2. The Center for Epidemiological Studies Depression Scale (CES-D)

The Center for Epidemiological Studies Depression (CES-D) scale was originally developed by Radloff in 1977 and has been widely used for depression measurement [87,88]. In the current study, the Chinese version of the CES-D was employed to assess depression among the participants [89,90,91]. This scale comprises 20 items, divided into three dimensions: “depressed affect (DA) (7 items)” (e.g., “I felt lonely” and “I felt sad”), “positive affect (PA) (4 items)” (e.g., “I felt hopeful about the future” and “I enjoyed life”) and “somatic complaints (SC) (9 items)” (e.g., “My appetite was poor” and “I was fearful”). DA and SC assess negative symptoms, and PA measures positive affect. Responses to items are scored on a Likert scale from 0 to 3 points (0 = rarely or less than 1 day a week, 1 = some of the time or 1–2 days a week, 2 = a moderate amount of the time or 3–4 days a week, and 3 = most or all of the times or 5–7 days a week), reflecting the frequency of symptoms experienced in the past week. The four items in positive affect dimension were reversely coded prior to formal analysis of data. A higher total score indicates a higher severity of depressive symptoms. Previous research has demonstrated the Chinese version of the CES-D scale’s robust internal consistency and construct validity in a sizable sample of Chinese adolescents [88,89,90,91,92,93], and it has been used to assess depression in Chinese children and young adolescents [88,89,92,93]. This three-factor structure showed good model fit in various studies conducted with Chinese early adolescents [90,92]. In the current research, the Cronbach’s α coefficient for the scale was 0.77, and the McDonald’s ω coefficient was 0.78, indicating high reliability.

### 2.3. Data Analyses

Independent sample t-tests were conducted to explore gender differences between paternal and maternal coparenting. Furthermore, network analysis was performed with RStudio (version 4.2.2). Firstly, network structures were constructed using the R-package qgraph [67,94]. The edges linking two nodes represented the regularized partial correlation coefficients between the parental coparenting network and young adolescents’ depression symptom network. Secondly, the strength and bridge strength of each node were calculated to clarify the significance of the nodes in the network. Higher values of the strength represent greater significance of nodes in the network, and higher bridge strength values for nodes indicate better capability to amplify activation of other network symptoms [78]. In the current network structure, the 11 nodes were categorized into two subnetworks, namely, the parental coparenting subnetwork and the depression symptom subnetwork. Finally, the robustness of the network was examined, including the accuracy of edge weights as well as the stability of strength and bridge strength of each node. To achieve this, the bootnet package in R was applied, which could examine the invariance of centrality indices by employing a bootstrap method to reduce the sample size in the network structure [95]. Furthermore, correlation stability coefficients (CS coefficients) were utilized to assess the stability of centrality indices, where a CS coefficient of 0.70 indicated the maximum acceptable reduction in sample size. CS coefficients above 0.50 were considered acceptable, with a minimum requirement of 0.25 [67,95]. 

## 3. Results

### 3.1. Descriptive Results

Table 1 presents the descriptive statistics results for paternal and maternal coparenting and depression in young adolescents. Regarding paternal and maternal coparenting, the independent sample *t*-tests revealed significant gender differences between boys and girls in their perception of coparenting across paternal and maternal integrity, conflict and disparagement dimensions, with all *p*-values falling below 0.05. Specifically, girls significantly perceived more paternal (*t* = 2.25, *p* < 0.05) and maternal integrity (*t* = 2.67, *p* < 0.05) than boys, whereas boys significantly perceived more paternal (*t* = −3.23, *p* < 0.05) and maternal conflict (*t* = −3.12, *p* < 0.05) as well as more paternal (*t* = −5.12, *p* < 0.05) and maternal disparagement (*t* = −4.20, *p* < 0.05) than girls. In terms of depressive symptoms, independent sample t-tests demonstrated a significant gender difference in somatic complaints, with boys exhibiting significantly higher scores than girls (*t* = 2.04, *p* < 0.05). Nevertheless, no significant gender differences were observed in the dimensions of depressed affect and positive affect, with all *p*-values exceeding 0.05. This suggested that boys experienced more negative coparenting and exhibited more somatic complaints in depressive symptoms than girls.

### 3.2. Network Analysis in the Total Sample of Adolescence

#### 3.2.1. Network Estimation

**Figure 1:**
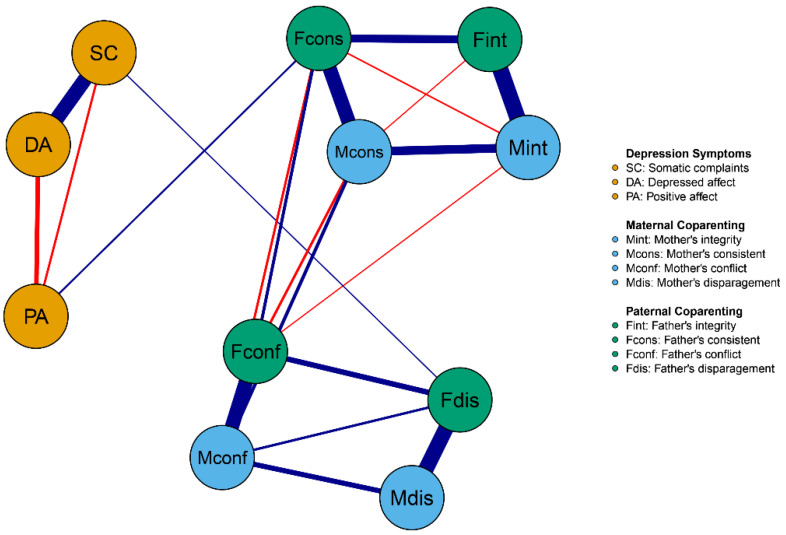
Network of coparenting and adolescent depression symptoms in the total sample (*N* = 793). Note. Nodes represent paternal and maternal coparenting and adolescents’ depression symptoms items; edges represent partial correlations between symptoms. Edge thickness indicates the strength of the partial correlations and edge color indicates the correlation valence (blue = positive, red = negative). Symptoms are shown in the same color (yellow = adolescent depression symptoms; sky-blue = maternal coparenting; green = paternal coparenting).

In order to examine the network structure of paternal and maternal coparenting on adolescents’ depressive symptoms, a normalized network with 11 nodes was estimated. There were 55 edges in total (11 × (11 − 1)/2), among which 40 had non-zero weights, with an average weight of 0.08, as depicted in Figure 1. Among all edges in this network, the top two strongest edges were found between Fcons (father’s consistency) and PA (positive affect), as well as Fdis (father’s disparagement) and SC (somatic complaints), with edge weights of 0.08 and 0.05, respectively. 

#### 3.2.2. Centrality Estimation

**Figure 2:**
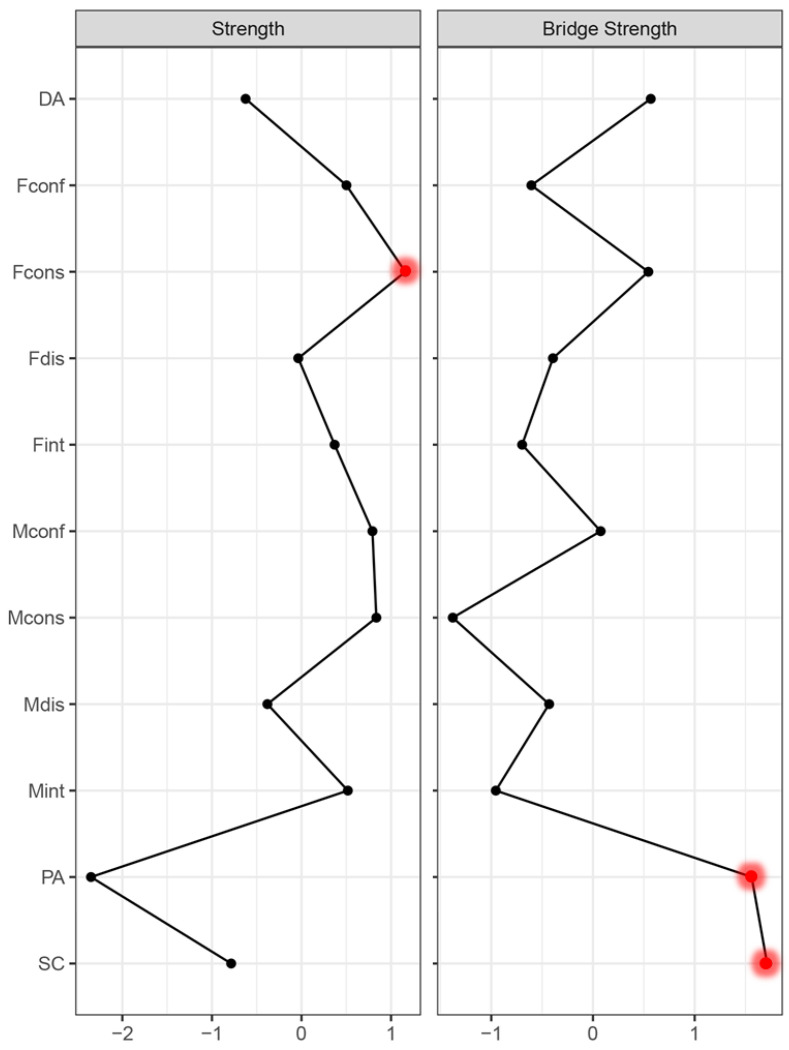
Strength and bridge strength of the network (*N* = 793). Note. Red (large) dots denote the most central symptoms.

As shown in Figure 2, Fcons (father’s consistency), Mcons (mother’s consistency) and Mconf (mother’s conflict), with respective strength values of 1.16, 0.84 and 0.79, were identified to present the highest node strength values. This suggested strong and close associations with other variables in the network. Additionally, as unveiled by bridge analysis, nodes with bridge strength values exceeding 1 included SC (somatic complaints) and PA (positive affect). Furthermore, concerning parental and maternal coparenting, Fcons (father’s consistency), Mconf (mother’s conflict) and Fdis (father’s disparagement) also exhibited notable bridge strengths, implying that they were crucial bridges linking the entire network. 

#### 3.2.3. Network Accuracy and Stability

For one thing, the results of edge-weight bootstrapping (Figure S1) indicated that the estimation of the entire network was highly accurate. For another, the stability test of the node centrality demonstrated robust stability in terms of strength (*CS* = 0.67, 95% *CI* (0.59, 0.75)) and bridge strength (*CS* = 0.75, 95% *CI* (0.67, 1.00)) (see Supplementary Material Figure S2). 

### 3.3. Networks in Girls and Boys

#### 3.3.1. Network Estimation

**Figure 3:**
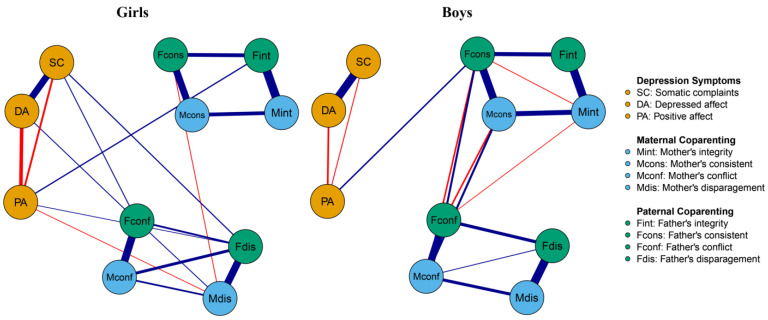
Network of coparenting parenting and adolescent depression symptoms in girls (*N* = 281) and boys (*N* = 512). Note. Nodes represent paternal and maternal coparenting and adolescents’ depression symptoms items; edges represent partial correlations between symptoms. Edge thickness indicates the strength of the partial correlations and edge color indicates the correlation valence (blue = positive, red = negative). Symptoms are shown in the same color (yellow = adolescent depression symptoms; sky-blue = maternal coparenting; green = paternal coparenting).

Given the significant differences observed between different gender groups regarding coparenting and depressive symptoms, this research further investigated the gender differences in network structure. As reflected in Figure 3, in the girls’ network, there were 31 edges with non-zero weights, with an average weight of 0.07. In the boys’ network, there were 37 edges with non-zero weights, with an average weight of 0.08. Notably, the most robust undirected edges between the paternal and maternal coparenting subnetwork and the adolescent depressive symptom subnetwork in the girls’ network were father’s integrity and positive affect (Fint-PA, edge weight = 0.10), father’s disparagement and somatic complaints (Fdis-SC, edge weight = 0.09), as well as father’s conflict and somatic complaints (Fconf-SC, edge weight = 0.08). In contrast, in the boys’ network, the most robust undirected edges were the father’s consistency and positive affect (Fcons-PA, edge weight = 0.12). 

#### 3.3.2. Centrality Estimation

**Figure 4:**
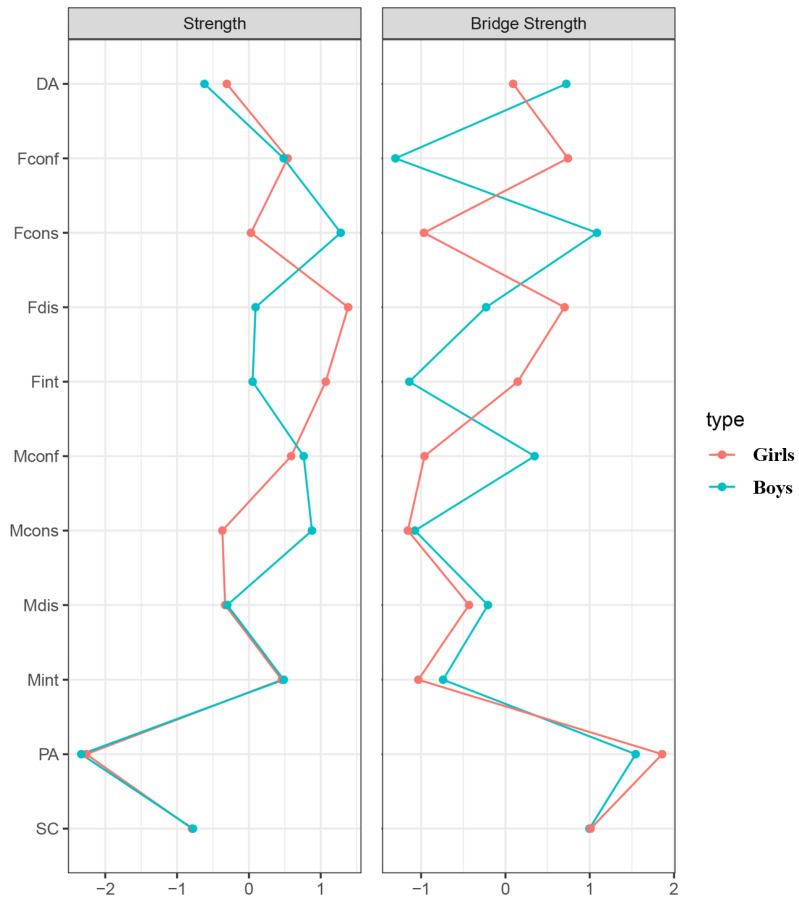
Standardized estimates of centrality for coparenting and adolescent depression symptoms by gender. Note. The red line represents girls, and the blue line represents boys.

As depicted in Figure 4, in the girls’ network, the nodes with a strength value greater than 1 were Fdis (father’s disparagement, strength = 1.38) and Fint (father’s integrity, strength = 1.07), whereas in the boys’ network, the node with a strength value greater than 1 was Fcons (father’s consistency, strength = 1.28). In both networks, nodes with bridge strength values greater than 1 were PA (positive affect) and SC (somatic complaints). Notably, in the boys’ network, Fcons (father’s consistency) also demonstrated a higher bridge strength, indicating that these nodes served as significant bridges linking the entire network.

#### 3.3.3. Network Accuracy and Stability

The results of edge-weight bootstrapping (Figure S3) indicate that the estimation of the networks for both boys and girls was highly accurate. The stability analysis (see Figure S4) manifested that in the girls’ network, the CS coefficient of strength was 0.52 (95% *CI* (0.44, 0.59)), and the CS coefficient of bridge strength was 0.75 (95% *CI* (0.67, 1.00)). In the boys’ network, the CS coefficient of strength was 0.67 (95% *CI* (0.59, 0.75)), and the CS coefficient of bridge strength was 0.75 (95% *CI* (0.67, 1.00)). These findings suggested good overall stability in both the boys’ and girls’ networks.

### 3.4. Network Comparison

Three tests were conducted to compare the gender differences in boys’ and girls’ networks. To begin with, the test for network structural invariance revealed no significant differences in the overall network structure across genders (M = 0.16, *p* = 0.602), suggesting a similarity in the overall structure among boys and girls. Secondly, the test for global strength invariance also showed no significant disparity in global network strength between genders (global strength for girls = 5.18, global strength for boys = 5.87, S = 0.69, *p* = 0.433). This confirmed a comparable network density in overall strength in both the boys’ and girls’ networks. Thirdly, the edge invariance test demonstrated significant differences in three edges between boys’ and girls’ networks. Notably, the edges linking Fdis (father’s disparagement) and PA (positive affect), as well as the one between Fint (father’s integrity) and PA (positive affect), exhibited greater strength in the girls’ networks compared to boys’ networks. In other words, relative to boys, positive affect in girls was more likely to be impacted by both paternal disparagement and paternal integrity. Details about all significant differences in edge invariance testing are provided as Supplementary Information (Table S1).

## 4. Discussion

Drawing on the family systems theory and the network theory of mental disorders, this research employed network analysis to probe into the relationship between parental and maternal coparenting and adolescent depressive symptom networks in Chinese young adolescents, and we further compared the gender differences in the networks of boys and girls. As can be seen from the results, within the dynamics of parental coparenting, boys tended to perceive more negative coparenting, whereas girls were more likely to experience a higher level of positive coparenting. Additionally, the results of network analysis indicated that the most central variables in the entire network consistently included Fcons (father’s consistency), Mcons (mother’s consistency) and Mconf (mother’s conflict) in the total sample for both genders. Moreover, Fcons (father’s consistency), Mconf (mother’s conflict) and Fdis (father’s disparagement) in coparenting, as well as SC (somatic complaints) and PA (positive affect) in depressive symptoms, exhibit high bridge strengths in the overall sample network. Furthermore, Fcons (father’s consistency) demonstrates a higher bridge strength in boys compared to girls. Despite the absence of significant differences in global network strength between girls and boys, specific edge connections vary between the genders. This provided a comprehensive and nuanced understanding of how parental coparenting affected depression in boys and girls and offered insights into targeted interventions for early adolescent depression by enhancing paternal and maternal coparenting. The following discussion will further explore several key findings.

To begin with, the descriptive statistical results revealed a significant gender difference. In the positive coparenting dimensions of paternal consistency and maternal consistency, girls exhibited significantly higher scores than boys. On the contrary, in negative coparenting dimensions encompassing paternal conflict, paternal disparagement, maternal conflict and maternal disparagement, boys consistently scored significantly higher than girls. On this basis, it can be inferred that boys tend to perceive more negative coparenting, whereas girls tend to perceive more positive coparenting from their parents. Previous research has found that parents are more likely to use gender-differentiated parenting when coparenting children [96,97]. In Chinese culture, parents exhibit marked differences in the gender role expectations assigned to young boys and girls [98]. In most Chinese families, males are typically expected to inherit the family surname and bear the responsibility of ensuring the family’s prosperity [98,99]. Hence, they are assigned gender expectations of decisiveness, resilience and independence. Under this context, parents frequently adopt harsh parenting strategies to fulfill their high expectations and socialization goals for boys [99,100]. Furthermore, impacted by these gender expectations for males, there is a perception that boys should possess greater resilience and stress resistance, thereby making parents tend to be less sensitive to the needs and emotional changes of boys. When parental conflicts arise due to differing opinions, there is often a lack of attention to avoiding the involvement of boys [101]. In the meantime, during the early stages of adolescence, boys often exhibit a strong rebellious mindset, daring to deviate from social norms or challenge parental and other authoritative figures. In view of this, when disciplining boys, parents may unintentionally undermine the authority of the coparent by asserting their own authoritative and irreplaceable position [30]. In stark contrast, unlike boys, Chinese girls are often socialized to be submissive, obedient and dependent [99]. They exhibit a greater tendency to adhere to parental opinions. As a result, in the upbringing of girls, parents place more emphasis on attending to their emotional needs and adopt a more consistent and cooperative approach to coparenting [30,42].

In addition, the results of network analysis indicated that the most central variables in the entire network consistently included Fcons (father’s consistency), Mcons (mother’s consistency) and Mconf (mother’s conflict) in the total sample for both genders. The consistent behavior in coparenting highlights the value of parents engaging in mutual communication, offering support to each other and jointly making decisions to reach a consensus on diverse realms, such as children’s participation in school matters and family activities, the establishment of peer relationships as they collaboratively raise their children [31,42]. The transition from late childhood to early adolescence is a critical phase in an individual’s journey from infancy to maturity, representing a key phase for accomplishing socialization [102]. During this stage, children’s self-awareness and independence undergo rapid development, fostering a strong desire for freedom and autonomy [102]. Nonetheless, given the lack of mature social cognition, there is an increased need for parents to supervise and provide guidance on their behavior [99,102]. Both parents need to actively participate in collaboratively establishing rules for adolescents, particularly concerning behavioral norms and peer interactions, and maintain effective communication and interaction during rule setting, achieving consensus. In this way, parents can serve as models and demonstrations for children [103] and, more importantly, significantly promote the early development of social skills and emotional regulation of their children in adolescence [104]. Meanwhile, the central role of mother’s conflict is in line with the findings of other research [30]. In the majority of Chinese households, the traditional family role distribution of “husband as breadwinner, wife as homemaker” designates mothers as the central coordinators of family activities [105]. In the upbringing of children, mothers play a vital role, and the continuous interaction with mothers establishes a deep and enduring mother–child attachment relationship [15]. This dynamic gives rise to a predominant influence of maternal behavior and emotional experiences on children’s psychological development outcomes [17,106]. Drawing upon the spillover hypothesis of family systems theory [57], when mothers engage in conflicts and convey hostility toward fathers in the presence of adolescents, the negative emotions and behaviors experienced by mothers during marital conflicts can spill over into the parent–child subsystem [107]. This spillover effect results in adolescents experiencing negative emotions and a sense of insecurity during interactions with their mothers, thus affecting the development of their psychological health [57,108].

It is noteworthy that Fcons (father’s consistency), Mconf (mother’s conflict) and Fdis (father’s disparagement) in coparenting, as well as SC (somatic complaints) and PA (positive affect) in depression, exhibit high bridge strengths in the overall sample. Moreover, Fcons (father’s consistency) demonstrates a higher bridge strength in boys compared to girls. Previous research has also identified the impact of fathers’ positive and negative coparenting behaviors on adolescents’ depression [35,82,109]. For instance, a longitudinal study suggested that fathers’ negative coparenting behaviors contribute to subsequent improvements in adolescent depression levels [109]. One plausible explanation is that active father involvement in parenting, coupled with forming an alliance with mothers in joint child-rearing, not only consolidates mothers’ marital satisfaction [110] but also notably enhances their positive emotions. This, in turn, prompts mothers to express more positivity in parenting, so as to lift the quality of mother–child relationships [111]. This results in adolescents perceiving increased positive emotional support from both parents, offering a buffering effect against adolescent depression [112]. Nevertheless, deliberate derogation of mothers by fathers in the presence of adolescents not only diminishes the authority of mothers but also reinforces an insecure mother–child attachment [57]. In the meantime, this heightened insecurity contributes to adolescents being more susceptible to developing depression [19]. In addition to the vital impact of both positive and negative paternal coparenting on children’s mental health, previous research has also underscored the role of fathers’ mentalizing, such as Reflective Functioning, Parental Insightfulness, and Mind–Mindedness, alongside father–child attachment processes and fathers’ emotional regulation, in enhancing children’s socio-emotional competencies and social adaptation [16,28,113,114,115]. Thus, future research could integrate these factors comprehensively to thoroughly explore the unique role of fathers in children’s development.

This also, to some extent, supports the crossover hypothesis of family systems theory [57], suggesting that emotional behaviors emerging from fathers in the coparenting subsystem can exert an impact on the functioning of the mother–child subsystem [57,116]. Furthermore, this study revealed that Fcons (father’s consistency) exhibited a higher bridge strength in boys compared to girls. This pattern is in alignment with the same-sex matching effect model, suggesting that fathers and mothers play a more influential role in shaping the developmental outcomes of same-sex children than those of opposite-sex children [117]. For instance, existing research uncovered a negative association between paternal strictness and internalizing problem behaviors in adolescent boys and a positive association with their general satisfaction, whereas paternal strictness showed no impact on internalizing problem behavior and general satisfaction among adolescent girls [118]. Given that Fcons (father’s consistency), Mconf (mother’s conflict) and Fdis (father’s disparagement) in coparenting among the present adolescent sample all showed higher bridge strengths, it is plausible that enhancing paternal consistent coparenting behavior, reducing maternal conflict and paternal disparagement coparenting behavior may have the most significant impact in alleviating depressive symptom in Chinese young adolescents. Nevertheless, it is necessary to replicate and further verify the present findings across broader and more diverse samples within different regions of China in future studies. Additionally, the results also demonstrated that the values of bridge strengths for SC (somatic complaints) and PA (positive affect) were high in depressive symptoms in the overall sample, suggesting that interventions targeting these two bridge nodes may more significantly reduce the impact of parental negative coparenting on early adolescents’ depressive symptoms, thereby improving the overall social functioning of young adolescents, which requires verification in further clinical interventions.

Another noteworthy finding was that, although the network global strength showed no significant difference between boys and girls, variations were observed in edge weights. Specifically, the connections between Fdis (father’s disparagement) and PA (positive affect), as well as Fint (father’s integrity) and PA (positive affect), were stronger in girls. In other words, the impact of paternal integrity and paternal disparagement coparenting behavior on positive emotions was more significant in young girls. These results partially align with the opposite-sex effect, suggesting a more substantial impact of parents on children of the opposite sex [117]. This effect has been identified in previous research exploring the correlation between positive parental coparenting behavior and psychological adjustment in adolescents [119]. This research enriched the existing literature through the application of network analysis to examine the association between paternal coparenting and adolescent depression, thereby offering a more comprehensive insight into how both positive and negative paternal coparenting influences young adolescents’ depressive symptoms [88]. Moreover, the results also underlined the increased vulnerability of young girls’ depression to both negative and positive coparenting behaviors from fathers, further suggesting the significance of fathers being attentive to their behavior during the coparenting process with their spouse, particularly when involved in coparenting with a girl [30]. Furthermore, the results also suggest that interventions focusing on increasing paternal integrity coparenting behavior and reducing paternal disparagement coparenting behavior may be especially beneficial in alleviating depressive symptoms in girls, with a particular emphasis on fostering girls’ positive emotions during clinical interventions.

Although the present study provided a comprehensive and nuanced understanding of how parental coparenting is linked to depression in young Chinese girls and boys, there are several limitations that need to be considered when interpreting our results. Firstly, as this study was a cross-sectional survey, we are unable to definitively establish whether positive and negative parental coparenting behaviors contribute to an increased risk of depression in youth or whether the presence of depression in youth alters the coparenting pattern. A longitudinal study that tracks parental coparenting both before and after the onset of youth depression, employing the cross-lagged panel network analysis method, would be beneficial in determining whether changes in coparenting precede the emergence of depression or occur subsequent to it. Such research would shed light on the causal mechanisms underlying the relationship between coparenting and youth depression. Secondly, parental coparenting and youth depression were assessed through adolescents’ self-reports. Although adolescent-reported parenting is commonly used [120], this method could be susceptible to social desirability bias and the subjective judgment of adolescents. In view of this, future research might employ multiformat (such as reported from a parental perspective) and multimethod (such as on-site observations or video scoring) assessment methods to more comprehensively measure parental coparenting and adolescents’ depression. Thirdly, participants in this study were exclusively drawn from a province in Northern China, raising concerns about the generalizability of results to diverse cultures and populations. Thus, cross-cultural and cross-group research is required to further examine the results of this study. Fourthly, the relationship between parental coparenting behavior and children’s depression could be mediated or moderated by other family factors (e.g., parental depression and anxiety, parental self-efficacy) [121,122], but the current study is primarily focused on the impact of parental coparenting behaviors on youth depression. Future studies could consider collecting data on these additional factors and utilize network analysis to test their potential mediating or moderating effects on the relationship between coparenting and adolescent depression. Fifthly, it is imperative to acknowledge that a child’s depression can be influenced by a multitude of interconnected systems. Drawing upon the family–peer system linkages theory [82,123], the school system (e.g., teacher–adolescent relationships) and the peer system (e.g., characteristics of friends) may interact with family dynamics, collectively affecting adolescent mental health. Moreover, biological factors, such as genetic susceptibility, neural development and hormonal fluctuations during puberty, may also contribute to the etiology of adolescent depression [124]. Consequently, future research ought to endeavor to examine the complex interplay between these diverse systems and biological elements in shaping adolescent mental health outcomes. Ultimately, while the present study identifies children’s perceptions of parental coparenting behavior as a potential therapeutic target for addressing adolescent depression, it is essential to recognize the necessity for further intervention research to rigorously validate and elaborate on our findings. Moreover, longitudinal investigations are warranted to ascertain the sustained impacts of interventions that incorporate coparenting dynamics, thereby enhancing the empirical foundation for evidence-based clinical practices in this domain.

## 5. Conclusions

In summary, the present study is the first study to investigate the relationship between parental coparenting and depression among Chinese young adolescents by employing network analysis. We found that boys tended to receive more negative coparenting, whereas girls were more likely to experience a higher level of positive coparenting. The results of network analysis revealed that father’s consistency and mother’s consistency as well as mother’s conflict were identified as the most central coparenting components in the network of total samples and father’s consistency, mother’s conflict and father’s disparagement in coparenting, as well as somatic complaints and positive affect in depression, exhibiting high bridge strengths in the overall sample network. Moreover, gender differences were also found in the comparison of boys’ and girls’ networks. The results indicated that father’s consistency demonstrates a higher bridge strength in boys compared to girls and the edges linking father’s disparagement and positive affect, as well as father’s integrity and positive affect, were stronger in girls’ than in boys’ networks, which indicated that the impact of paternal integrity and paternal disparagement coparenting behavior on positive emotions is more significant in young girls. The present study contributes to our understanding of the relationship between parental coparenting and Chinese young adolescents’ depression from a network analysis perspective and uncovers potential implications for designing intervention and prevention strategies dedicated to improving adolescents’ depression through parental coparenting behaviors.

## Figures and Tables

**Table 1:** Descriptive results of paternal and maternal coparenting and young adolescents’ depression.

Variable	Abbr.	All Sample (*N* = 793)	Girls (*N* = 281)	Boys (*N* = 512)	*t*	*p*	Cohen’s d
		M (SD)	M (SD)	M (SD)			
Coparenting Behaviors							
Father’s integrity	Fint	32.48 ± 11.38	33.70 ± 11.41	31.80 ± 11.31	2.25	0.025	0.17
Father’s consistent	Fcons	45.96 ± 15.33	47.28 ± 15.31	45.23 ± 15.31	1.81	0.071	0.13
Father’s conflict	Fconf	15.84 ± 9.89	14.32 ± 9.73	16.68 ± 9.89	−3.23	0.001	0.24
Father’s disparagement	Fdis	14.51 ± 10.26	12.12 ± 9.24	15.82 ± 10.56	−5.12	<0.001	0.37
Mother’s integrity	Mint	32.28 ± 11.59	33.76 ± 11.56	31.47 ± 11.53	2.67	0.008	0.20
Mother’s consistent	Mcons	45.31 ± 16.40	45.64 ± 17.16	45.12 ± 15.98	0.42	0.678	0.03
Mother’s conflict	Mconf	15.53 ± 9.96	14.05 ± 9.57	16.35 ± 10.09	−3.12	0.002	0.23
Mother’s disparagement	Mdis	14.56 ± 10.40	12.53 ± 9.84	15.68 ± 10.53	−4.20	<0.001	0.31
Adolescents Depression Symptoms							
Somatic complaints	SC	0.91 ± 0.56	0.86 ± 0.57	0.94 ± 0.56	−2.04	0.042	0.15
Depressed affect	DA	0.94 ± 0.69	0.93 ± 0.71	0.95 ± 0.68	−0.45	0.655	0.03
Positive affect	PA	1.84 ± 0.69	1.83 ± 0.69	1.84 ± 0.69	−0.19	0.851	0.01

Note: Abbr. = abbreviation, M = mean, SD = standard deviation.

## Data Availability

The data that support the findings of this study are available from the corresponding author upon reasonable request.

## Supplementary Materials

The following supporting information can be downloaded at: https://www.mdpi.com/article/10.3390/bs14040297/s1, Figure S1: Edge-Weight Accuracy for Coparenting Parenting and Adolescents Depression Symptoms Network in Total Sample; Figure S2: Centrality Stability for Coparenting Parenting and Adolescents Depression Symptoms Network in Total Sample; Figure S3: Edge-Weight Accuracy for Coparenting Parenting and Adolescents Depression Symptoms Networks in Girls and Boys; Figure S4: Centrality Stability for Coparenting Parenting and Adolescents Depression Symptoms Networks in Girls and Boys; Table S1: The Permutation Test of Differences in Edges Weights.
