# Supplementary material for: How Coparenting Is Linked to Depression among Chinese Young Girls and Boys: Evidence from a Network Analysis
Source: Behav Sci (Basel). 2024 Apr 4;14(4):297. doi: 10.3390/bs14040297 (PMC11047583; doi:10.3390/bs14040297)
Supplement: Supplementary file 1 [file behavsci-14-00297-s001.zip › behavsci-2885235-supplementary.pdf]

Supplementary Materials

Additional Supporting Information may be found in the online version of this article.

Figure S1: Edge-weight accuracy for coparenting parenting and adolescent depression symptom network in total sample.

Figure S2: Centrality stability for coparenting parenting and adolescent depression symptom network in total sample.

Figure S3: Edge-weight accuracy for coparenting parenting and adolescent depression symptom networks in girls and boys.

Figure S4: Centrality stability for coparenting parenting and adolescent depression symptom networks in girls and boys.

Table S1: The permutation test of differences in edge weights.

Figure S1

Edge-weight accuracy for coparenting parenting and adolescent depression symptom network in total sample.

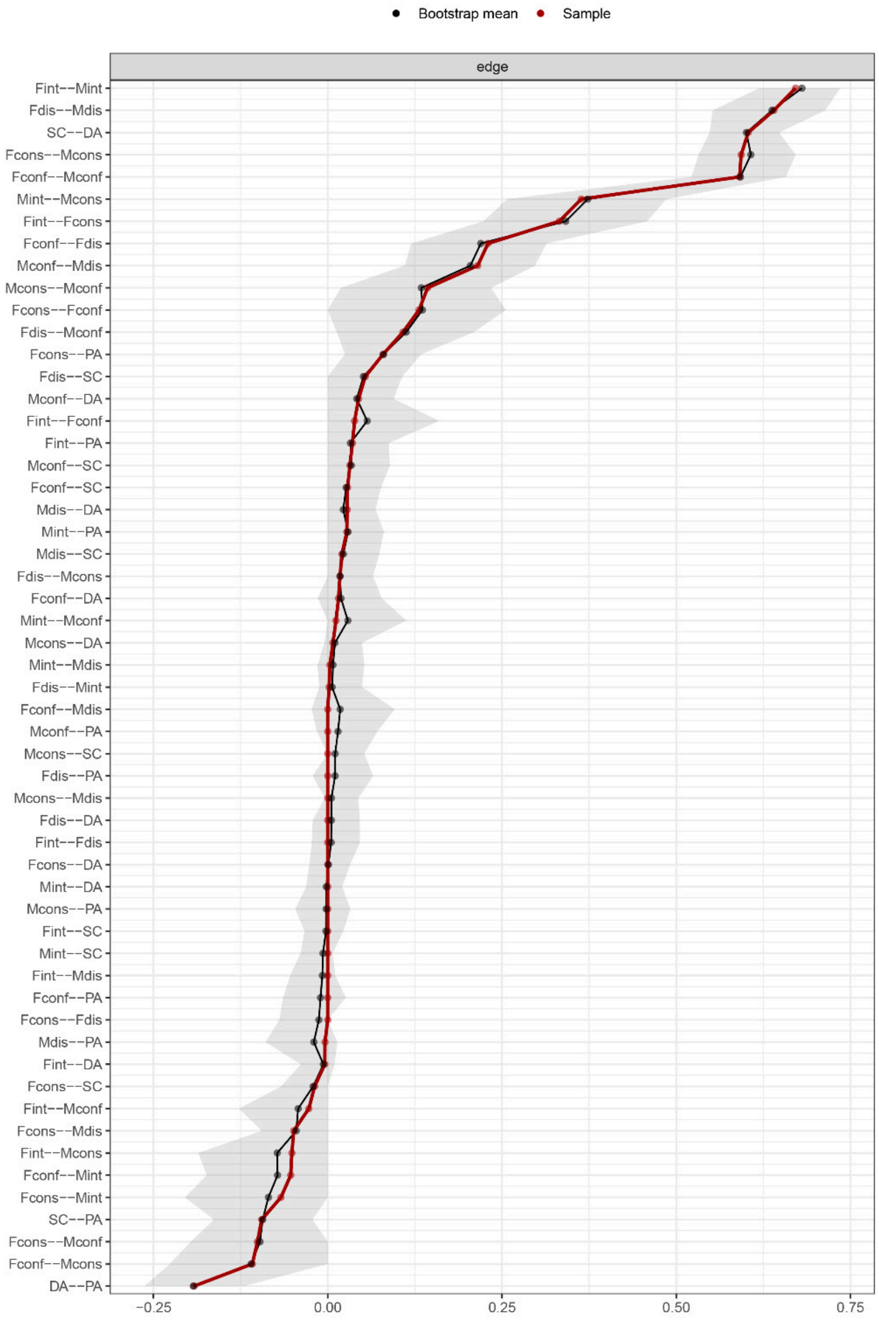

Note. Bootstrapped confidence intervals of estimated edge weights for the estimated network. The red line indicates the sample values, and the gray area indicates the bootstrapped confidence intervals. Each horizontal line represents one edge of the network, ordered from the edge with the highest edge weight to the edge with the lowest edge weight.

Figure S2

Centrality stability for coparenting parenting and adolescent depression symptom network in total sample.

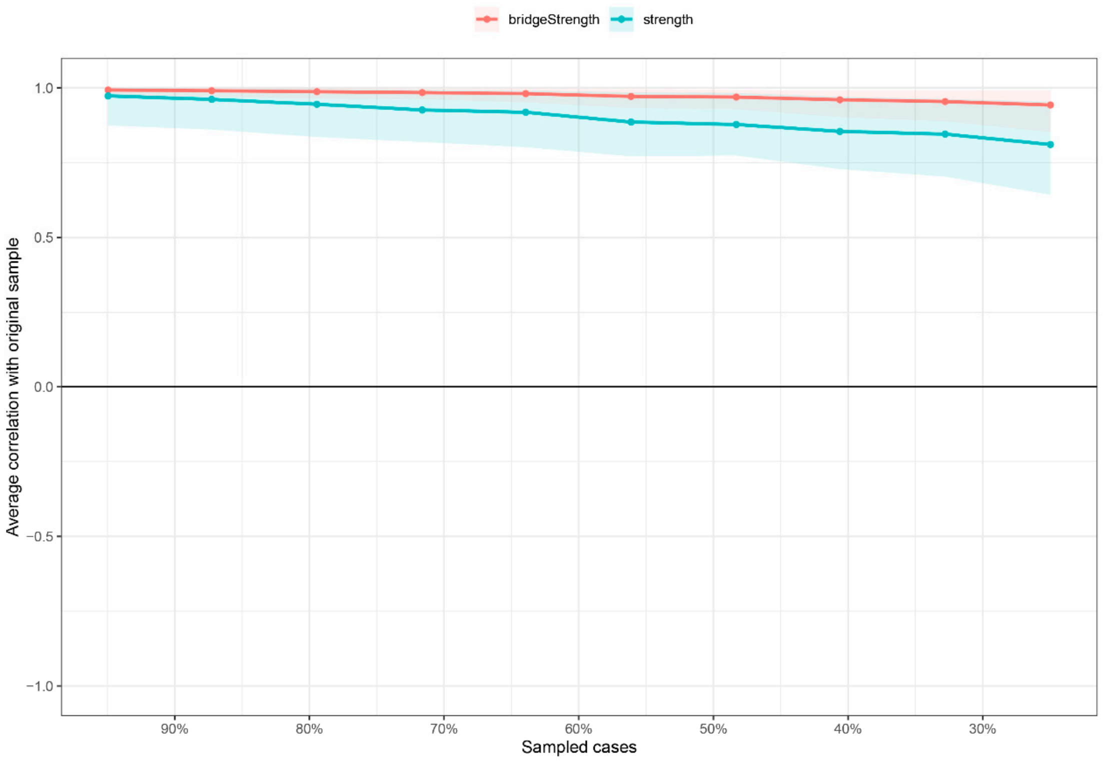

Note. Average correlations between centrality indices of networks sampled with persons dropped and the original sample. Lines indicate the means, and areas indicate the range from the 2.5th quantile to the 97.5th quantile.

Figure S3

Edge-weight accuracy for coparenting parenting and adolescent depression symptom networks in girls and boys.

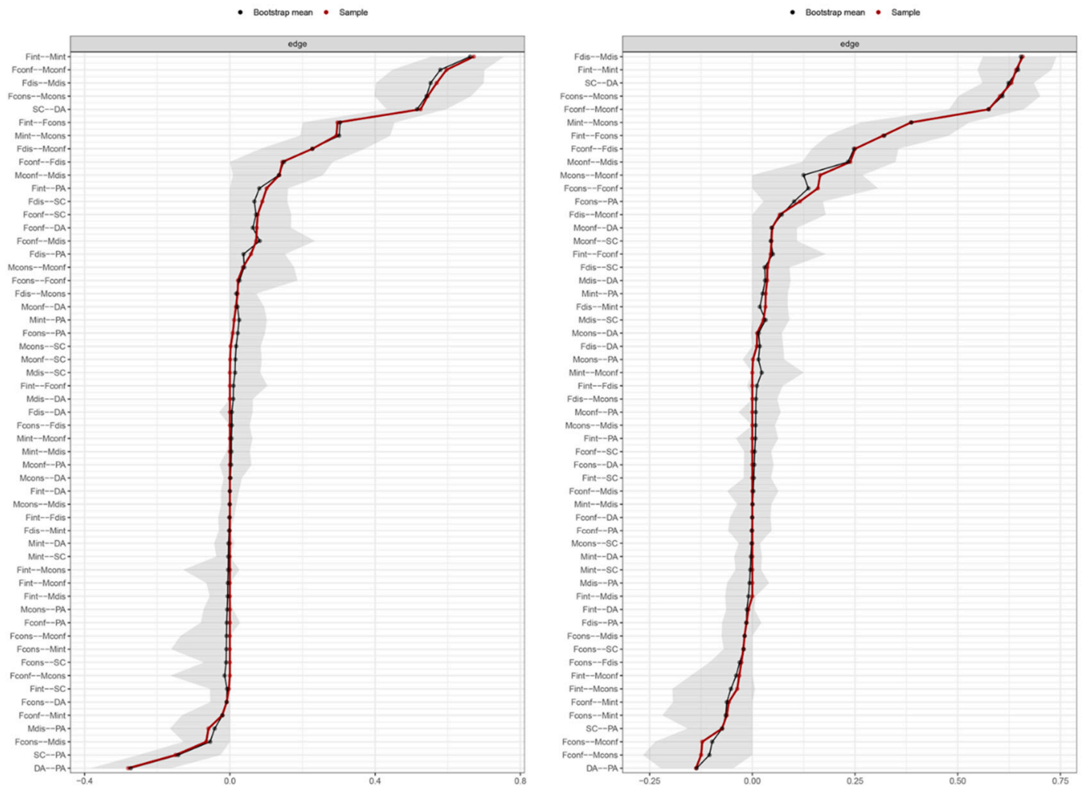

Note. Girls (left) and boys (right) in adolescence. Bootstrapped confidence intervals of estimated edge weights for the estimated network. The red line indicates the sample values, and the gray area indicates the bootstrapped confidence intervals. Each horizontal line represents one edge of the network, ordered from the edge with the highest edge weight to the edge with the lowest edge weight.

Figure S4

Centrality stability for coparenting parenting and adolescent depression symptom networks in girls and boys.

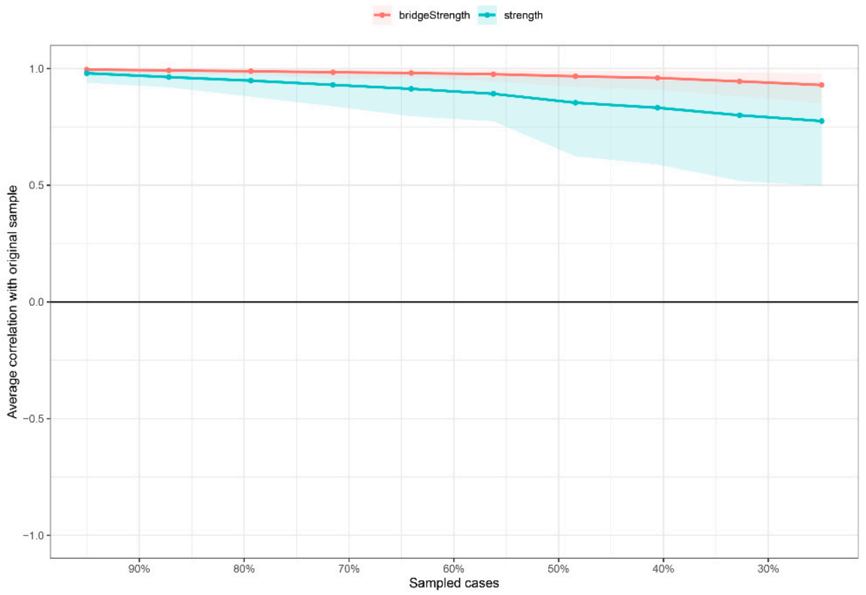

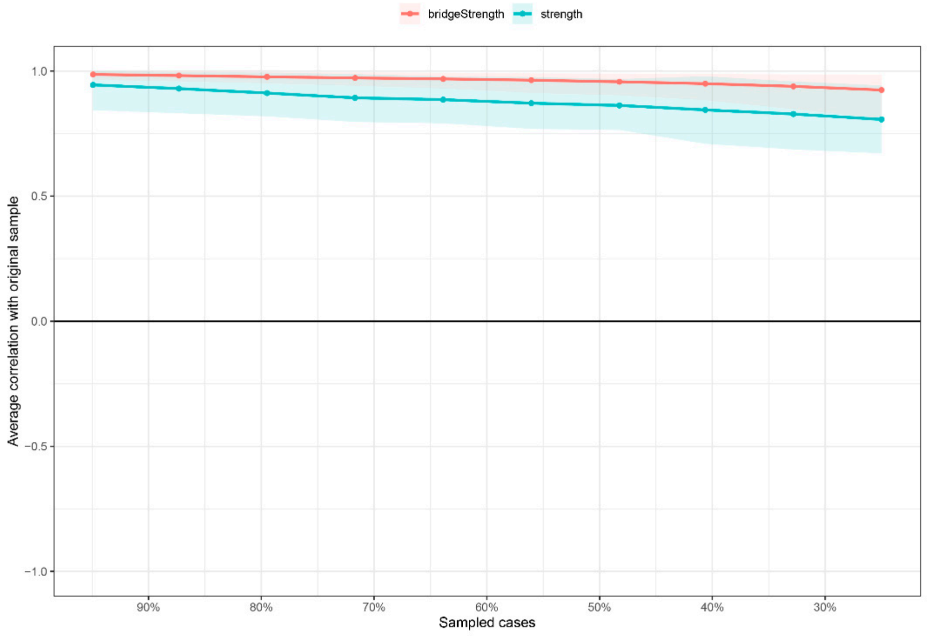

Note. Girls (upper) and boys (lower). Average correlations between centrality indices of networks sampled with persons dropped and the original sample. Lines indicate the means, and areas indicate the range from the 2.5th quantile to the 97.5th quantile.

Table S1 The permutation test of differences in edge weights (presenting only at $p < 0.05$)

Var1	Var2	*p*-value	Test statistic *E*
Fdis	PA	0.024	0.07
Fint	PA	0.027	0.10
Fdis	Mint	0.040	0.03
